# Supplementary material for: Temporal controls on silicic acid utilisation along the West Antarctic Peninsula
Source: Nat Commun. 2017 Mar 13;8:14645. doi: 10.1038/ncomms14645 (PMC5355812; doi:10.1038/ncomms14645)
Supplement: Supplementary Information — Supplementary Table, Supplementary Discussion, Supplementary Figure and Supplementary References [file ncomms14645-s1.pdf]

Supplementary Table 1: $\delta^{30}Si_{diatom}$ and $Si(OH)_4$ utilisation data from ODP Site 1098A, Palmer Deep, west Antarctic Peninsula.

Leg	Site	H	C	S	Half	Top (cm)	Bottom (cm)	NIGL.ref	Age (kyr)	$\delta^{30}Si_{diatom}$	% SI(OH)$_4$ utilisation
178	1098	A	1	1	W	10	10.5	CA168	0.24	+0.29	7.8
178	1098	A	1	2	W	30	30.5	CA174	0.73	+0.54	28.6
178	1098	A	2	1	W	20	20.5	CA177	1.43	+0.69	40.7
178	1098	A	2	1	W	80	80.5	CA180	1.65	+0.53	27.2
178	1098	A	2	1	W	121	121.5	CA165	1.81	+0.38	15.3
178	1098	A	2	2	W	50	50.5	CA184	2.10	+0.65	37.1
178	1098	A	2	2	W	70	70.5	CA185	2.17	+0.69	40.9
178	1098	A	2	2	W	130	130.5	CA10	2.39	+0.24	3.5
178	1098	A	2	3	W	40	40.5	CA190	2.60	+0.76	46.9
178	1098	A	2	3	W	80	80.5	CA192	2.74	+0.35	12.8
178	1098	A	2	3	W	120	120.5	CA194	2.88	+0.60	32.9
178	1098	A	2	4	W	10	10.5	CA11	3.02	+0.33	10.8
178	1098	A	2	4	W	110	110.5	CA197	3.37	+0.61	34.3
178	1098	A	2	4	W	130	130.5	CA198	3.44	+0.70	41.8
178	1098	A	2	5	W	40	40.5	CA15	3.64	+0.72	43.2
178	1098	A	2	6	W	10	10.5	CA205	4.05	+0.93	61.0
178	1098	A	2	6	W	110	110.5	CA19	4.38	+0.69	40.7
178	1098	A	2	6	W	129.5	130.0	CA20	4.45	+0.49	23.8
178	1098	A	2	7	W	59	59.5	CA208	4.72	+0.62	35.3
178	1098	A	3	1	W	10	10.5	CA209	4.86	+0.76	47.0
178	1098	A	3	1	W	50	50.5	CA211	4.99	+0.61	34.1
178	1098	A	3	1	W	130	130.5	CA215	5.26	+0.39	15.4
178	1098	A	3	2	W	80	80.5	CA218	5.60	+0.80	49.8
178	1098	A	3	2	W	140	140.5	CA221	5.81	+0.93	60.7
178	1098	A	3	3	W	50	50.5	CA224	6.01	+1.00	67.0
178	1098	A	3	3	W	70	70.5	CA225	6.08	+0.87	55.4
178	1098	A	3	3	W	130	130.5	CA228	6.29	+0.82	52.0
178	1098	A	3	4	W	20	20.5	CA230	6.43	+0.52	26.8
178	1098	A	3	4	W	60	60.5	CA232	6.57	+0.78	48.3
178	1098	A	3	4	W	100	100.5	CA233	6.71	+0.64	37.0
178	1098	A	3	5	W	10	10.5	CA236	6.93	+0.88	56.7
178	1098	A	3	5	W	69.5	70	CA29	7.15	+0.95	62.1
178	1098	A	3	5	W	90	90.5	CA237	7.22	+1.03	69.4
178	1098	A	3	6	W	20	20.5	CA240	7.52	+0.96	63.3
178	1098	A	3	6	W	40	40.5	CA241	7.59	+0.96	63.6
178	1098	A	4	1	W	10	10.5	CA246	8.10	+0.73	44.0
178	1098	A	4	1	W	30	30.5	CA247	8.18	+0.78	48.0
178	1098	A	4	1	W	90	90.5	CA250	8.42	+0.78	48.3
178	1098	A	4	2	W	18.5	19	CA35	8.73	+0.66	38.4
178	1098	A	4	2	W	60	60.5	CA252	8.90	+0.75	46.1
178	1098	A	4	2	W	80	80.5	CA253	8.99	+0.37	14.5
178	1098	A	4	6	W	120	120.5	CA283	9.11	+0.81	50.7
178	1098	A	5	1	W	70	70.5	CA290	9.98	+0.76	46.7
178	1098	A	5	1	W	110	110.5	CA292	10.14	+0.68	40.3
178	1098	A	5	2	W	40	40.5	CA296	10.29	+0.70	41.9
178	1098	A	5	2	W	100	100.5	CA39	10.36	+0.68	39.8
178	1098	A	5	3	W	69	69.5	CA301	10.49	+0.69	40.4
178	1098	A	5	4	W	40	40.5	CA307	10.62	+0.79	48.8
178	1098	A	5	4	W	100	100.5	CA310	10.69	+1.15	79.4
178	1098	A	5	4	W	120	120.5	CA311	10.71	+0.66	38.7
178	1098	A	6	1	W	50	50.5	CA46	11.92	+0.81	50.8
178	1098	A	6	1	W	110.8	111.4	CA55	12.09	+0.63	36.1
178	1098	A	6	2	W	124.1	124.3	CA88	12.55	+0.82	51.5

Supplementary Discussion

To date, the only comparable $\delta^{30}Si_{diatom}$ record to the one presented here from ODP Site 1098 along the Antarctic coastal margin comes from Adélie Land, East Antarctica[1]. Whilst the two locations are characterised by different oceanographic and atmospheric conditions, a comparison remains useful given the likelihood for future studies on Holocene biogeochemical cycling and rates of nutrient utilisation along the coastal Antarctic margin.

The significantly higher values of $\delta^{30}Si_{diatom}$ at ODP Site 1098 relative to Adélie Land reflects differences in the isotopic composition of source waters supplied to the photic zone and levels of silicic productivity (Supplementary Figure 1). Nonetheless, Holocene patterns of $\delta^{30}Si_{diatom}$ at both sites show little similarity. Through the early Holocene (Zone 2b) the relative stability of values at ODP Site 1098 is contrasted by a significant decline in $\delta^{30}Si_{diatom}$ at Adélie Land from c. +0.6‰ to c. 0.0‰ that is concordant with sea-ice expansion and water column stratification in the East Antarctic region[1]. In contrast to the interpretation of the $\delta^{30}Si_{diatom}$ data at ODP Site 1098, enhanced stratification at Adélie Land is argued to deplete the concentration of nitrate and other nutrient in the water column, leading to a net reduction in rates of silicic acid utilisation[1].

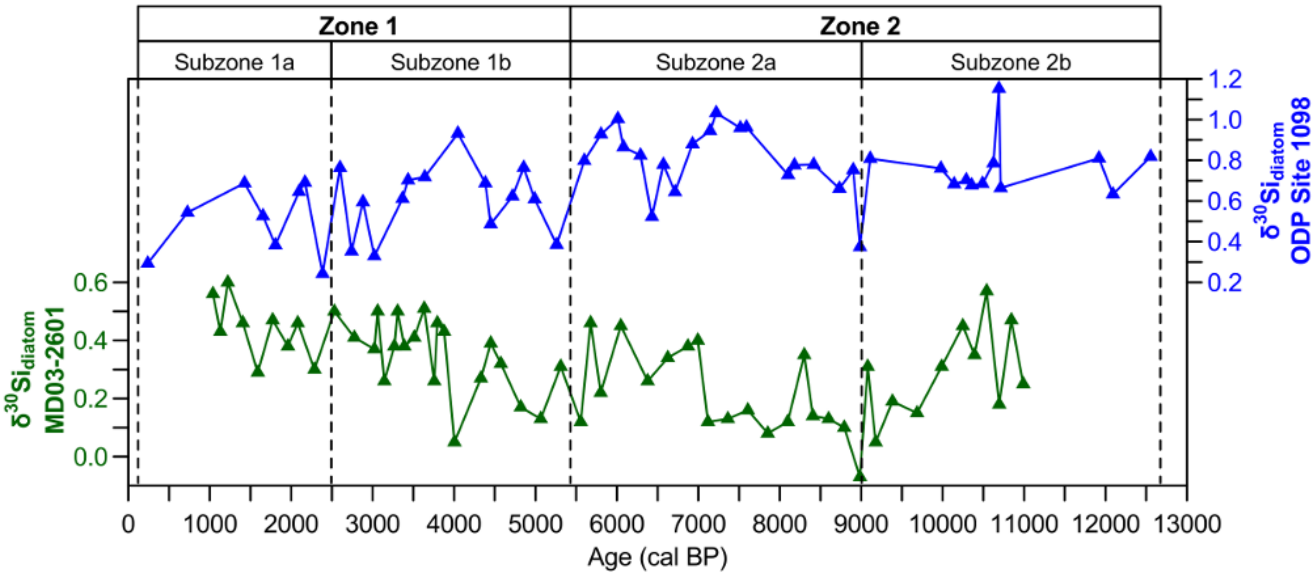

Supplementary Figure 1: Comparison of $\delta^{30}Si_{diatom}$ from ODP Site 1098 along the West Antarctic Peninsula (blue) and $\delta^{30}Si_{diatom}$ from MD03-2601 off Adélie Land, East Antarctica (green). Zones are those defined in the manuscript for the $Si(OH)_4$ utilisation record at ODP Site 1098.

Whilst the resolution of both records is insufficient to make robust comparisons though the mid-Holocene, encompassing subzones 2a and 1b, both sites display fluctuations of 0.2-0.6‰ reiterating the potential for significant changes in biogeochemical cycling along the Antarctic coastal margin. However, whereas the neoglacial interval along the west Antarctic Peninsula (WAP) is characterised by a prolonged shift towards lower $\delta^{30}Si_{diatom}$ and rates of $Si(OH)_4$ utilisation linked in part to increased glacial discharge, opposite trends are observed at Adélie Land[1]. Of note is the decoupling of $\delta^{30}Si_{diatom}$ and diatom carbon isotope record ($\delta^{13}C_{diatom}$) at Adélie Land over this interval. Whilst the mechanics behind this remain unresolved, the decoupling may

indicate iron fertilisation linked to increased glacial discharge and/or increases in the relative proportion of frustules living within sea-ice[1]. Along the WAP issues of Fe fertilisation can be discounted due to the lack of iron limitation and the replenishment instead of photic zone iron concentrations via deep winter mixing[2,3,4]. Similarly, as argued in the main text, cryophilic diatoms represent only a minor component of the analysed samples at ODP Site 1098.

Supplementary References

1 Panizzo, V. *et al*. Sea ice diatom contributions to Holocene nutrient utilization in East Antarctica. *Paleoceanography* **29,** 328-342 (2014).

2 Ardelan, M.V. *et al*. Natural iron enrichment around the Antarctic Peninsula in the Southern Ocean. *Biogeosciences* **7,** 11-25 (2010).

3 Huang, K., Ducklow, H., Vernet, M., Cassar, N., Bender, M.L. Export production and its regulating factors in the West Antarctica Peninsula region of the Southern Ocean. *Global Biogeochem. Cy.* **26,** GB2005, doi:10.1029/2010GB004028 (2012).

4 Annett, A.L. *et al*. Comparative roles of upwelling and glacial iron sources in Ryder Bay, coastal western Antarctic Peninsula. *Mar. Chem.* 176, 21-33 (2015).
